# Comorbidities of Adolescent Pregnancy at a Tertiary Teaching Hospital in Southwestern Uganda

**DOI:** 10.7759/cureus.109957

**Published:** 2026-05-31

**Authors:** Onesmus Byamukama, Charles Tushabomwe-Kazooba, Michael Kanyesigye, Annah Amwikirize, Arnold Kamugisha, Moses Ntaro, Henry Ochola, Joseph Ngonzi

**Affiliations:** 1 Obstetrics and Gynecology, Mbarara University of Science and Technology, Mbarara, UGA; 2 Administration, Mbarara University of Science and Technology, Mbarara, UGA; 3 Biostatistics and Epidemiology, Mbarara University of Science and Technology, Mbarara, UGA; 4 Community Health, Mbarara University of Science and Technology, Mbarara, UGA

**Keywords:** adolescent pregnancies, co-morbidities, teaching hospital, tertiary, uganda

## Abstract

Background

Adolescent pregnancy presents significant health challenges worldwide. Each year, millions of teenage girls in developing regions become pregnant. In Uganda, teenage pregnancy remains common, with marked regional variations. Adolescents in low socioeconomic situations, with limited education, or who face barriers to contraceptive access are more prone to unintended pregnancies. These pregnancies may result in multiple health complications, including premature births, small for gestational age babies, postpartum depression, anaemia, and urinary tract infections (UTIs).

Methods

A cross-sectional study was conducted at the tertiary institution of Mbarara Regional Referral Hospital, Mbarara, Uganda, from July to September 2024, including adolescent pregnant women who presented for antenatal care (ANC) attendance or delivery. Data collection involved clinical examinations (blood pressure and mid-upper arm circumference (MUAC)) and laboratory tests (haemoglobin, HIV, hepatitis B, random blood sugar, and urinalysis). Data on socio-demographic and obstetric characteristics were collected through a structured questionnaire. Data were analysed using STATA version 17 (StataCorp LLC, College Station, TX, USA), with comorbidities defined by clinical criteria such as hypertension, anaemia, HIV infection, pre-eclampsia, hyperglycaemia, and UTIs.

Results

Of the total, 252 participants were enrolled. The median age was 18.5 years. Most were married (81.7%), rural residents (54.5%), had completed primary education (52%), and had not used modern contraceptives (84.5%). Among participants, 10.4% were in arranged marriages, 31% had unplanned pregnancies, and 4% had engaged in transactional sex. The average ANC attendance was four visits. Notably, 99.6% of participants had at least one comorbidity, and 3.2% had two or more. The most common comorbidities included UTIs (53.0%), anaemia (34.8%), and HIV infection (8.7%). Less common conditions included hepatitis B (0.4%), hyperglycaemia (7.4%), and pre-eclampsia (1.2%).

Conclusion

Adolescent pregnancies show a high prevalence of comorbidities, particularly UTIs, anaemia, and HIV. Improved screening and management of these conditions could reduce maternal and neonatal morbidity and mortality.

## Introduction

Adolescent pregnancy is associated with substantial health and socio-economic challenges [1,2]. Adolescent pregnancy describes pregnancy in girls between 10 and 19 years of age [1]. In developing regions, approximately 21 million teenage girls become pregnant every year, and nearly 12 million of these pregnancies result in childbirth [3]. Sub-Saharan Africa (SSA) continues to have a high rate of 99.4 births per 1000 women [4]. Uganda’s teenage pregnancy rate, estimated at 25%, is among the highest in SSA; however, the burden is not evenly distributed, varying from 16% to 31% [5-7].

Adolescent pregnancies are common among the uneducated and the poor, those who had child marriages or experienced child sexual abuse, as well as those who face barriers to obtaining and using contraceptives to prevent unintended pregnancies [8-10]. Early coitus initiation, as well as the decreasing age at menarche, which affects a woman’s fertility, all contribute to adolescent pregnancy, which remains a serious social, economic, and health problem [11].

Adolescent births result in health consequences such as preterm births, small for gestational age, and increased neonatal death, while women might experience mental health conditions, hypertensive disorders of pregnancy, sepsis, and death [11-14]. Adolescent mothers also face social and economic consequences; for example, school dropouts, low socio-economic status, and poor growth and development for their children [15].

Adolescent pregnancies are also associated with obstetric co-morbidities like anaemia, urinary tract infections (UTIs), anogenital infections, hepatitis, preterm pre-labour membrane rupture, low-lying placenta, gestational diabetes, and preeclampsia [16-19]. Poor outcomes in adolescent pregnancy result from both the biological vulnerabilities of young maternal age and the influence of socio-demographic factors [20]. However, some of these pregnancy co-morbidities are due to gynaecological immaturity and poor nutritional reserves of the mother. It has been shown that the interaction between biological, social, and economic factors is responsible for the observed outcomes of these adolescent pregnancies [20].

Previously conducted studies in our setting have described the burden and risk factors for adolescent pregnancy. However, there is a paucity of data on the comorbidities among this high-risk group of pregnant women. Despite adolescent pregnancy being common in Uganda, the clinical burden of co-existing conditions among pregnant adolescents remains poorly described, limiting the ability of antenatal services to provide targeted screening, early treatment, and risk-based care for this vulnerable group. This study determined the prevalence and described the spectrum of co-morbidities among adolescent pregnant women attending Mbarara Regional Referral Hospital in southwestern Uganda.

## Materials and methods

Study site, design, and population

A cross-sectional study was undertaken from July 2024 to September 2024 at the maternity ward and Maternal and Child Health Clinic of Mbarara Regional Referral Hospital in Mbarara City, southwestern Uganda. The hospital serves as the main referral facility for the southwestern region and also acts as a teaching hospital for Mbarara University of Science and Technology. Annually, the department records approximately 11,000 deliveries, about 1,000 of which are among adolescent mothers, representing a rate of ~10%. The antenatal clinic runs Monday to Friday and attends to approximately 50 pregnant adolescent women. The hospital has a level three laboratory equipped to perform all the tests recommended during antenatal care (ANC).

Our study population was adolescent pregnant women who were admitted for delivery or sought care at the ANC clinic of Mbarara Regional Referral Hospital. We excluded any woman who could not recall her date of birth and had no national identification card, which could be used to capture the date of birth. Participants were recruited consecutively from both the antenatal clinic and maternity ward/delivery admissions.

Sample size and sampling technique

We used the Kish-Leslie formula (1965) for cross-sectional studies, $n = \frac{z^2pq}{d^2}$, where z = the critical value (it is 1.96 at a 0.05 level of significance), d = error margin of 5%, and p = prevalence.

A study conducted in a hospital in Nepal identified UTI as the primary outcome, with a prevalence of 18.4% [21]. Using the formula for sample size calculation for a single proportion, a sample size of 230 participants was determined. After adjusting for a 10% non-response rate, a total of 252 participants were considered. We consecutively enrolled mothers in the maternity ward and ANC clinic who met the inclusion criteria.

This study was approved by the Mbarara University of Science and Technology Research Ethics Committee (Ref No. MUST 09/05-17), and administrative clearance was obtained from Mbarara Regional Referral Hospital. Written informed consent was obtained from participants aged 18 years and above. For participants younger than 18 years, assent was obtained from the adolescent participant, together with consent from a parent/guardian or legally authorised representative, in accordance with ethical requirements for research involving minors.

Data sources and study variables

Data collection was conducted by two research assistants, both midwives, using structured interviewer-administered questionnaires after training in the study procedures and data collection tool (Appendix 1).

This study was conducted at the antenatal clinic of Mbarara Regional Referral Hospital and involved adolescent pregnant women, defined as those from 10 to 19 years of age. The participants were recruited during an ANC visit when seeking care. The participants were selected consecutively until the sample size was attained. The participants gave both verbal and written consent. Women who declined consent were not included in the study. The participants were also subjected to an interviewer-guided questionnaire in a language they best understood to collect socio-demographic and obstetric characteristics. Physical examination was done to determine blood pressure, presence or absence of pallor of the mucous membranes, mid-upper arm circumference (MUAC), weight, height, and symphysio-fundal length. Three millilitres of blood were drawn by venepuncture at the cubital fossa of the non-dominant upper limb and put in a purple-top vacutainer. The participant also submitted a midstream, clean-catch urine specimen. Both samples were transported at room temperature (25°C) to the MRRH clinical laboratory for haemoglobin estimation, *Treponema pallidum* haemagglutination assay (TPHA), hepatitis B surface antigen (HBsAg), random blood sugar, HIV testing, and urinalysis (dipstick and microscopy). A copy of the results was given to the participant, and all those who had abnormal results were linked to the high-risk obstetrics clinic for further care. The research team also entered the results into the participants’ data collection tool.

Our outcome variable was the presence of any one of the tested comorbidities. In this study, comorbidities were defined as any concurrent clinically relevant medical, infectious, nutritional, metabolic, or obstetric condition identified among adolescent pregnant women during ANC or delivery assessment, including UTI, anaemia, HIV infection, hepatitis B, syphilis, hyperglycaemia, undernutrition, and pre-eclampsia. The comorbidities of interest include UTIs (considered present if either dipstick test positive for leucocyte esterase and/or nitrite, or the presence of pyuria on microscopy, or both) [22,23]; anaemia (haemoglobin level less than 11.0 g/dL) [24,25]; pre-eclampsia/eclampsia (presence of high blood pressure - systolic BP ≥ 140 mmHg and diastolic BP ≥ 90 mmHg - plus proteinuria on urine dipstick); hyperglycaemia in pregnancy (random blood sugar > 11.0 mmol/L); syphilis (TPHA seropositivity); hepatitis B (HBsAg seropositivity); undernutrition (MUAC < 23 cm); and HIV infection.

The questionnaire captured independent variables related to socio-demographic and obstetric factors. The socio-demographic characteristics assessed were age, marital status, parity, residence status (rural versus urban), occupation, religion, level of education, and partner support. Obstetric and medical factors included gestational age, type of pregnancy, gestational age at booking for ANC, initiation of iron/folic acid supplementation, sulfadoxine-pyrimethamine (SP) (Fansidar) prophylaxis, and chronic medical illnesses (hypertension and diabetes mellitus).

All laboratory tests were conducted in the Mbarara Regional Referral Hospital clinical laboratory following standard operating procedures. Internal quality control procedures were performed according to routine laboratory protocols, including the use of appropriate control materials where applicable. Laboratory equipment was routinely maintained and calibrated as part of the hospital’s quality assurance system to ensure reliability and reproducibility of results.

Bias

Various measures were adopted to limit potential sources of bias in the study. Selection bias was reduced by consecutively recruiting all eligible pregnant adolescent women attending MRRH during the study period. Information bias was minimised through standardised data collection procedures, including structured questionnaires administered by trained research assistants and laboratory-based confirmation of comorbidities. Recall bias was mitigated by collecting data from participants at the point of care, reducing reliance on retrospective self-reports. Additionally, data entry and analysis were conducted using double-checking mechanisms to prevent systematic errors.

Data management and analysis

After entry into REDCap, the data were exported to Stata version 17 (StataCorp, College Station, TX, USA) for analysis. Descriptive statistics for demographic and obstetric characteristics were presented as frequencies and percentages.

We summarised maternal sociodemographic and obstetric characteristics as frequencies and percentages. The prevalence of comorbidities among adolescent pregnant women was calculated as the proportion of those with comorbidities, expressed as a percentage. We described the comorbidities on a bar graph with frequencies and percentages. We assessed missing data and performed a complete-case analysis.

Ethical considerations

This study was approved by the Mbarara University of Science and Technology Research Ethics Committee (Ref No. MUST 09/05-17), and administrative clearance was obtained from Mbarara Regional Referral Hospital. Written informed consent was obtained from participants aged 18 years and above. For participants younger than 18 years, assent was obtained from the adolescent participant, together with consent from a parent/guardian or legally authorised representative, in accordance with ethical requirements for research involving minors.

## Results

**Figure 1:**
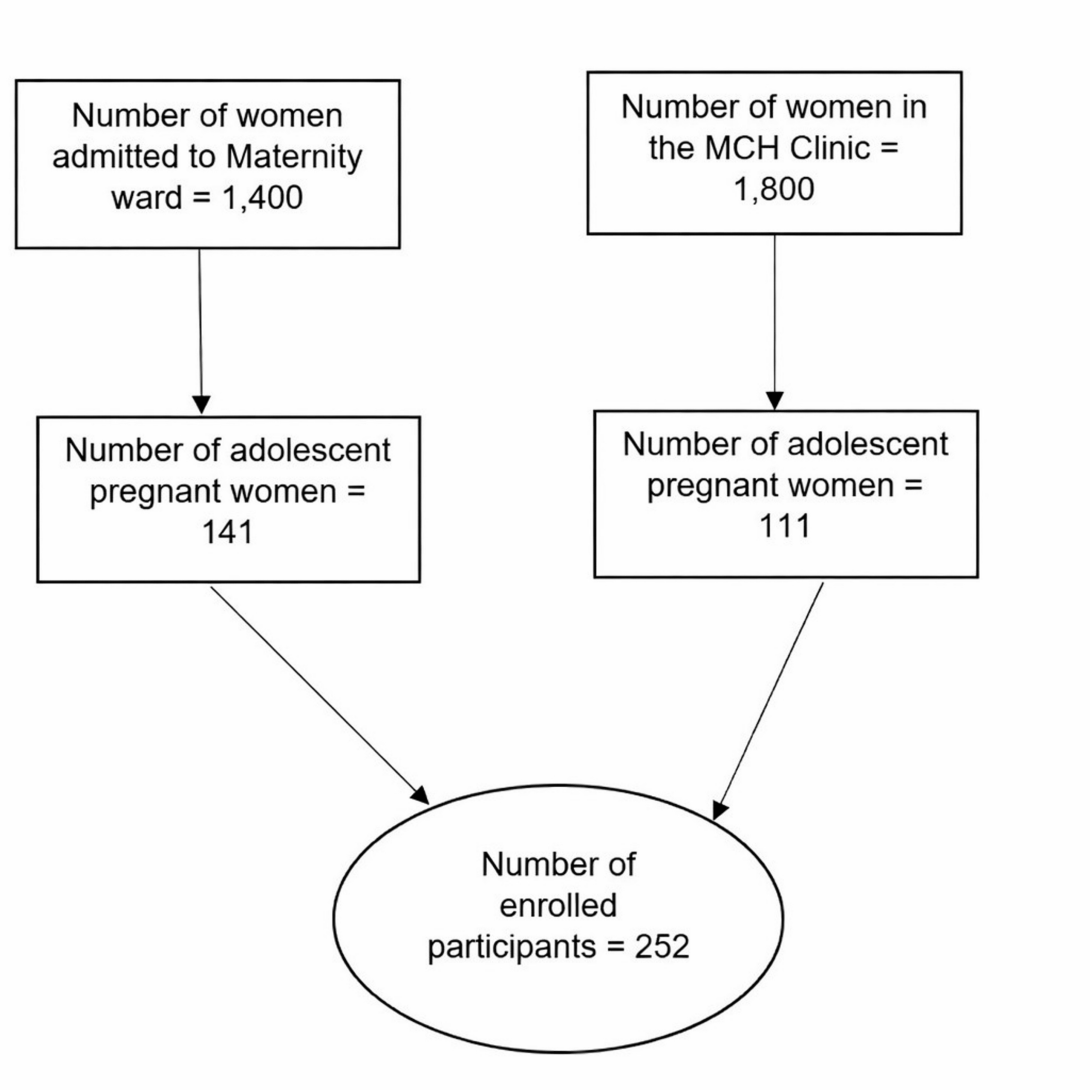
Participant recruitment

We present results for 252 participants (adolescents) who were enrolled in the study. No pregnant adolescent woman was excluded from the study, and there were no missing data (Figure 1).

Socio-demographic characteristics of study participants

A total of 252 participants were enrolled. The median age was 18.5 (18-19) years, and the median monthly income was 30,000 (10,000-60,000). Most participants were married (81.7%), rural residents (54.5%), had completed primary education (52%), and were unemployed (50.8%). Among participants, 10.4% were in arranged marriages, and 4% had engaged in transactional sex (Table 1).

**Table 1:** Socio-demographic characteristics of adolescent pregnant women at Mbarara Regional Referral Hospital *Monthly income in Ugandan shillings IQR, interquartile range

Characteristics	Total (N = 252)
Residence type	
Urban	115 (45.6%)
Rural	137 (54.4%)
Age in years, median (IQR)	18.5 (18-19)
Marital status	
Unmarried	45 (18.3%)
Married	206 (81.7%)
Level of Education	
None/Did not complete primary education	76 (30.2%)
Completed primary education	131 (52.0%)
Completed at least secondary education	45 (17.9%)
Occupation	
None/Peasant farmer	128 (50.8%)
Business/professional	114 (45.2%)
Student	10 (4.0%)
Monthly income, median (IQR) *	30000 (10000-60000)
Age in years at first sexual intercourse, median (IQR)	17 (16-17)
Number of sexual partners, median (IQR)	1 (1-2)
Type of marriage arrangement	
Arranged by parents/relatives	26 (10.4%)
Met the partner by myself	225 (89.6%)
Ever engaged in transactional sex	
No	242 (96.0%)
Yes	10 (4.0%)

Obstetric characteristics of study participants

Of the 252 participants, the median gravidity was 1, and the median gestational age was 38 weeks (IQR 29-39), with a median ANC attendance of 4 visits (IQR 3-5). The majority had not used modern contraceptives, 213/252 (84.5%), and 78/252 (31%) had unplanned pregnancies (Table 2).

**Table 2:** Obstetric characteristics of adolescent pregnant women enrolled in the study *Parity refers to deliveries above 26 weeks of gestational age. IQR, interquartile range

Obstetric characteristics	Total (N = 252)
Gravidity, median (IQR)	1 (1-1)
Parity*, median (IQR)	0 (0-1)
Gestational age in completed weeks, median (IQR)	38 (29-39)
ANC attendance, median (IQR)	4 (3-5)
Was this pregnancy planned	
No	78 (31.0%)
Yes	174 (69.0%)
Use of modern contraceptive methods	
Yes	39 (15.5%)
No	213 (84.5%)

Prevalence of co-morbidities among adolescent pregnant women

**Figure 2:**
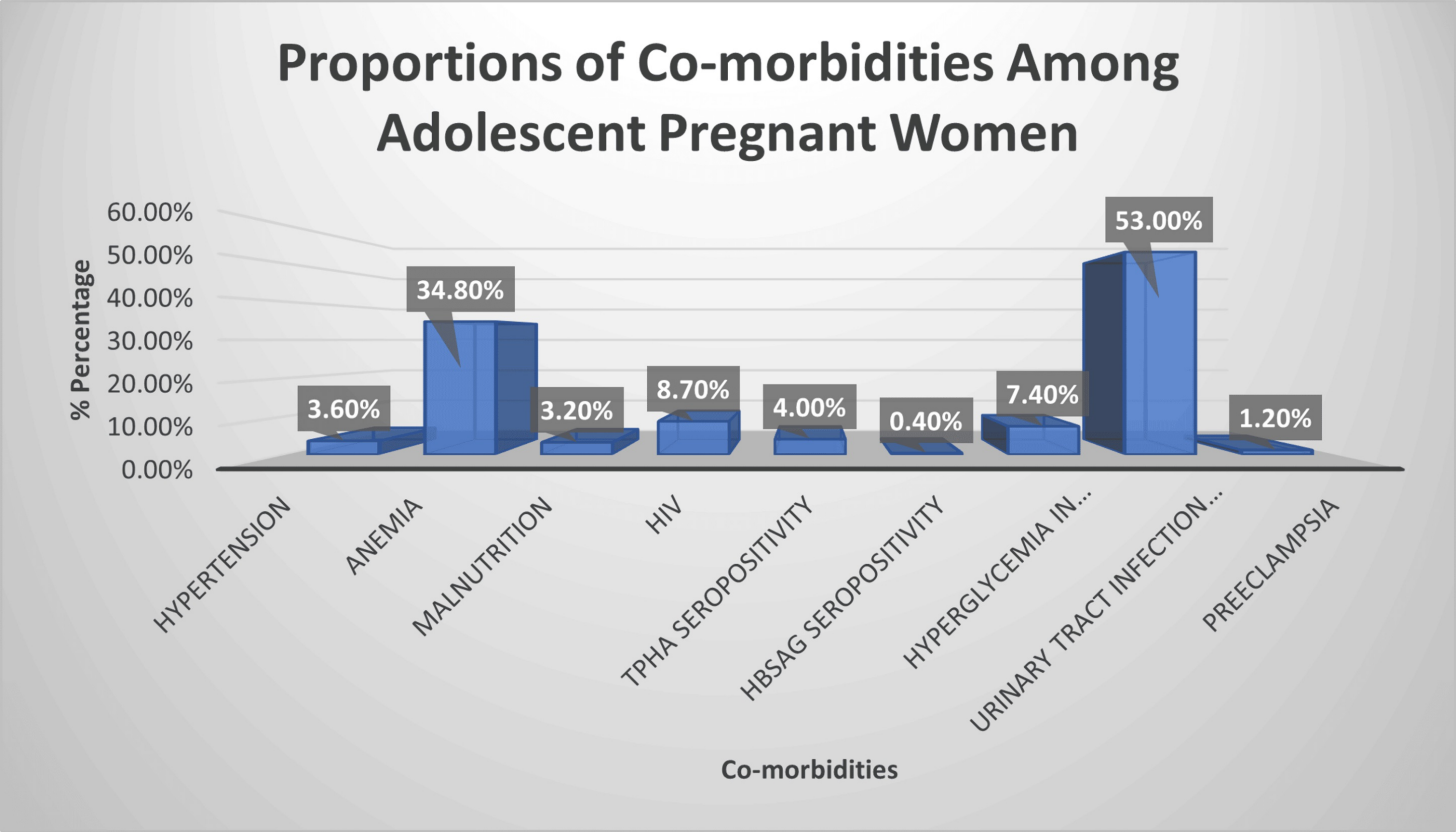
Graph showing the proportions of co-morbidities among adolescent pregnant women

Of the 252 participants who were enrolled in the study, almost all, 251/252 (99.6%), had at least one comorbidity, and 3.2% had two or more. The most common comorbidities included urinary tract infections (UTIs), 132/252 (53.0%); anaemia, 86/252 (34.8%); HIV infection, 22/252 (8.7%); and hyperglycaemia, 18/252 (7.4%). Less common conditions included hepatitis B seropositivity (0.4%) and pre-eclampsia (1.2%) (Figure 2).

## Discussion

This study investigated the prevalence and spectrum of comorbidities among adolescent pregnant women attending Mbarara Regional Referral Hospital, southwestern Uganda. Over 99% of participants had at least one comorbidity, with UTI (53%), anaemia (34.8%), and HIV (8.7%) being the most prevalent conditions.

The high prevalence of UTIs aligns with prior research indicating high susceptibility in this age group due to hormonal and anatomical factors influencing urinary tract function during pregnancy [26,27]. Anaemia was the second most common comorbidity (34.8%), aligning with studies from SSA, where nutritional deficiencies, recurrent infections, and lack of ANC contribute significantly to poor haemoglobin levels during pregnancy [28-30]. Similarly, HIV prevalence (8.7%) among study participants underscores the intersection of adolescent pregnancy and existing public health challenges in Uganda.

The finding that nearly all participants had at least one comorbidity is consistent with previous literature suggesting that adolescent pregnancy carries heightened biological and socio-economic risks [30]. The interplay of factors such as limited access to healthcare services, nutritional deficiencies, and the physiological immaturity of adolescents exacerbates these risks [27]. For example, pre-eclampsia, observed in 1.2% of the cohort, has been strongly linked to younger maternal age, underlining the urgent need for early screening and management [7,8].

This study adds to the growing body of evidence on the challenges faced by adolescent mothers in low-resource settings. Notably, we observed a low prevalence of pre-eclampsia and hepatitis B compared to studies in similar populations, which may reflect regional variations or differences in diagnostic criteria and healthcare access [11,30].

Strengths and limitations

Our study's strengths include its focus on a vulnerable population within a tertiary teaching hospital, ensuring comprehensive data collection on a wide range of comorbidities. The use of laboratory confirmation for diagnoses strengthens the validity of our findings. Nevertheless, the study’s cross-sectional design limits conclusions about causality between socio-demographic factors and comorbidities. Additionally, our reliance on self-reported data for some variables may introduce recall bias. UTI was defined based on urinalysis findings, including leukocyte esterase, nitrites, and/or pyuria. Urine culture was not performed; therefore, the reported UTI prevalence may include asymptomatic laboratory abnormalities and may overestimate the prevalence of culture-confirmed UTI. The high overall prevalence of comorbidities should be interpreted with caution because some conditions, particularly UTI, were defined using screening laboratory findings rather than confirmatory culture. This may have resulted in overestimation of true infection prevalence. Additionally, the findings should be interpreted as reflecting the burden among adolescents accessing referral-level care rather than the general adolescent pregnant population.

Implications for practice and policy

These findings highlight the critical need for tailored ANC programs targeting adolescent mothers. Interventions should prioritise early identification and treatment of UTIs, anaemia, and HIV, alongside robust nutritional support and counselling. Furthermore, integrating HIV services with ANC could mitigate the impact of the high prevalence of HIV in this group. Policymakers should also address systemic barriers to healthcare access for adolescents, particularly in rural settings where socio-economic vulnerabilities are pronounced.

Future research opportunities

Prospective studies should be undertaken to explore the long-term risks to adolescent mothers and their infants. Further research should investigate the interplay of hormonal and anatomical factors, such as changes in the vaginal mucosa, that predispose adolescents to comorbidities like UTIs. Additionally, qualitative research examining the experiences of adolescent mothers in accessing healthcare services could inform the development of more adolescent-friendly policies and practices.

Generalisability

The findings of this study are generalisable to adolescent pregnant women receiving care at tertiary hospitals in southwestern Uganda and similar low-resource settings. However, as the study was conducted in a referral hospital, the prevalence of comorbidities may be higher than in community-based populations. Differences in healthcare access, socioeconomic factors, and cultural practices may limit the direct applicability of these results to adolescents in other regions. Further multi-centre studies are needed to enhance external validity.

## Conclusions

Adolescent pregnant women in southwestern Uganda experience a very high burden of comorbidities, with UTIs, anaemia, and HIV being the most common conditions identified. Improved screening and management of common comorbidities among adolescent pregnant women may support earlier identification of health risks and inform targeted ANC strategies, although longitudinal studies are needed to determine their effect on maternal and neonatal outcomes. Addressing the broader social and health-system barriers faced by adolescent mothers will be critical to reducing maternal and neonatal morbidity and improving pregnancy outcomes in this high-risk population.

## Appendices

Appendix 1

Study Number: ___________

Hospital IP Number: ____________

Date of enrolment: ___________

Site of Enrollment

○ ANC ○ Maternity

Demographic Data

District: ___________

Category of Residence

○ Rural ○ Urban

What is your age? ___________

○ Date of birth ___________

○ Participant doesn't know the date of birth

System age ___________

What is your marital status?

○ Single

○ Married/cohabiting

○ Separated/divorced

What is your religious affiliation?

○ Catholic

○ Anglican

○ Moslem

○ Others

Other religion ___________

What is your level of education?

○ None/Did not complete primary education

○ Completed Primary education

○ Completed Secondary education

○ Completed Tertiary education

What is your occupation?

○ None

○ Business

○ Peasant

○ Farmer

○ Student

○ Professional

○ Others

Other occupation ___________

How much do you earn per month? (UGx) ___________

Age at first sexual intercourse ___________

Number of sexual partners ___________

Number of sexual partners the partner has? ___________

○ Dont know the sexual partners

History of use of modern contraceptive methods

○ Yes

○ No

If Yes, Which ones

○ Pills

○ Implants

○ Injectables

○ IUCD

○ Condoms

○ Others

Other contraceptives used ___________

Knowledge about PreP

○ Yes

○ No

Have you used PreP before?

○ Yes

○ No

Type of marriage arrangement

○ Arranged by parents/relatives

○ Met the partner by myself

Circumstances around conception

○ Consensual sexual intercourse

○ Forced/raped

Knew sexual partners HIV status before sexual encounter

○ Yes

○ No

Obstetric Factors

Gravidity ___________

Parity (Delivered above 26 weeks) ___________ (Enter 999: for first pregnancy)

No. of previous abortions ___________ (Enter 0: if no abortions)

LNMP (dd/mm/yyyy) ___________

○ Don't know LNMP

Gestational age in (completed weeks) ___________

How many times did you attend ANC ___________

Number of living children (Only for parous women) ___________

Gestational age at ANC booking in (months) ___________

Interventions during ANC (Tick all)

○ HBSAg test ○ TPHA test ○ HB Estimation ○ HIV test ○ RBS ○ Folic acid ○ Iron tablets

Was this pregnancy planned?

○ Yes

○ No

Have u ever engaged in transactional sex?

○ Yes

○ No

If yes, was this pregnancy a result of transactional sex?

○ Yes

○ No

Hospital Evaluation

Blood Pressure

Systolic ___________ Diastolic ___________

HDP present

○ Yes

○ No

HB estimated (g/dL) ___________

MUAC (cm) ___________

HIV status

○ Negative

○ Positive

○ Unknown

HBSAg

○ Negative

○ Positive

○ Not done

TPHA

○ Negative

○ Positive

○ Not done

Random blood sugar (RBS) ___________

Urinalysis

Leucocytes ___________

Proteins ___________

Nitrities 

○ Negative

○ Positive

Pus cells ___________

Urinary tract infection present (UTI)

○ Yes

○ No

Date_created

○ Yes

○ No

Entered by ___________

Appuser ___________
